# Cubilin expression is monoallelic and epigenetically augmented via PPARs

**DOI:** 10.1186/1471-2164-14-405

**Published:** 2013-06-18

**Authors:** Obaidullah Aseem, Jeremy L Barth, Sandra C Klatt, Brian T Smith, W Scott Argraves

**Affiliations:** 1 Department of Regenerative Medicine and Cell Biology, Medical University of South Carolina, Charleston, SC, 29425, USA

**Keywords:** Cubilin, Megalin, LRP-2, Amnionless, Albumin, Epigenetic, Monoallelic Expression, CpG Island, DNA Methylation, 5-Azacytidine, 5Aza, Trichostatin A, TSA, Histone Hypoacetylation, Histone Deacetylase, HDAC, Peroxisome Proliferator-Activated Receptor, PPAR, Kidney, Proximal Tubule, Intestine, Enterocyte

## Abstract

**Background:**

Cubilin is an endocytic receptor that is necessary for renal and intestinal absorption of a range of ligands. Endocytosis mediated by cubilin and its co-receptor megalin is the principal mechanism for proximal tubule reabsorption of proteins from the glomerular filtrate. Cubilin is also required for intestinal endocytosis of intrinsic factor-vitamin B_12_ complex. Despite its importance, little is known about the regulation of cubilin expression.

**Results:**

Here we show that cubilin expression is under epigenetic regulation by at least two processes. The first process involves inactivation of expression of one of the cubilin alleles. This monoallelic expression state could not be transformed to biallelic by inhibiting DNA methylation or histone deacetylation. The second process involves transcriptional regulation of cubilin by peroxisome proliferator-activated receptor (PPAR) transcription factors that are themselves regulated by DNA methylation and histone deacetylation. This is supported by findings that inhibitors of DNA methylation and histone deacetylation, 5Aza and TSA, increase cubilin mRNA and protein in renal and intestinal cell lines. Not only was the expression of PPARα and γ inducible by 5Aza and TSA, but the positive effects of TSA and 5Aza on cubilin expression were also dependent on both increased PPAR transcription and activation. Additionally, 5Aza and TSA had similar effects on the expression of the cubilin co-receptor, megalin.

**Conclusions:**

Together, these findings reveal that cubilin and megalin mRNA expression is under epigenetic control and thus point to new avenues for overcoming pathological suppression of these genes through targeting of epigenetic regulatory processes.

## Background

Cubilin is a 460-kDa peripheral membrane glycoprotein, anchored to the plasma membrane via an amino terminal amphipathic helix [1,2] and through interactions with the transmembrane protein, amnionless, forming the so-called cubam complex [3,4]. Cubilin is expressed by the absorptive epithelia of tissues such as renal proximal convoluted tubules [5], ileum [6], and yolk sac [7], where it mediates the endocytosis of numerous ligands [1], in some cases acting in concert with another endocytic receptor, LRP-2/megalin [8].

Cubilin was first described as the intrinsic factor-cobalamin/vitamin B_12_ receptor important for intestinal absorption of vitamin B_12_[9]. Mutations of the cubilin gene are the cause of Imerslund-Gräsbeck syndrome, also known as selective vitamin B_12_ malabsorption with proteinuria [10]. Proteinuria in these individuals results from the inability of the cubilin-deficient kidney to reabsorb ligands that filter across the glomerulus, including albumin and apolipoprotein A-I, the major apolipoprotein of HDL [11,12].

In vitro studies have shown that cubilin mRNA expression is stimulated by retinoic acid and that it is not sterol-regulated [8]. Cubilin is co-expressed with megalin in numerous tissues [7,8,13,14], suggesting the possibility that the genes share common transcriptional regulatory mechanisms. Peroxisome proliferator-activated receptors (PPARs), transcription factors belonging to the nuclear receptor superfamily, upregulate megalin expression [15], but it is not yet known whether these factors influence cubilin expression. Furthermore, expression of megalin is regulated by histone acetylation and methylation and DNA methylation [16], but it is not yet known whether cubilin is regulated epigenetically in a similar manner.

The availability of a mouse carrying a knockin of an EGFP cassette into the cubilin gene [17] has enabled precise histological analysis of cubilin expression. While evaluating cubilin-EGFP expression in renal tissue of mice heterozygous for the knockin allele, we observed a striking difference in the distribution of EGFP immunoreactivity versus cubilin immunoreactivity. Although both were detected at sites that matched cubilin distribution in wildtype animals, the cellular distributions in heterozygous mice were largely exclusive such that cells appeared to express predominantly either EGFP or cubilin. This led us to postulate that cubilin might undergo an allelic inactivation that silenced or strongly diminished expression of either the maternal or paternal allele. This phenomenon, called monoallelic expression [18], can occur through chromosomal inactivation (e.g., X inactivation), autosomal gene imprinting or random gene inactivation [19]. Monoallelic expression of a variety of autosomal genes have been described [19,20], including p120 catenin [21], certain cytokines [22], olfactory receptors and antigen receptors [23,24]. Here we explored the possibility that *cubilin* (Cubn), an autosomal gene [25], is regulated through epigenetic mechanisms and whether such processes might have consequences on cubilin function and on the expression of its partners, amnionless and megalin.

## Results

### Monoallelic expression of cubilin in the renal proximal tubules

**Figure 1:**
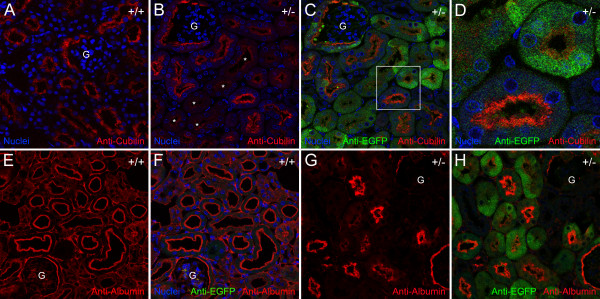
**Monoallelic expression of cubilin in *Cubn***^***+/del exon 1–6;EGFP***^**mice leads to reduced albumin binding to brush borders of cubilin-deficient renal proximal tubules cells. A** shows a confocal micrograph of a section of wildtype mouse kidney labeled with anti-cubilin IgG (*red*) and the nuclear stain, Draq5. **B** shows a confocal image from a section of a *Cubn*^*+/del exon 1–6;EGFP*^ mouse kidney labeled with anti-cubilin and Draq5. *Asterisks* indicate proximal tubules displaying relatively low levels of anti-cubilin immunolabeling. **C** and **D** are merged images of anti-cubilin immunolabeling (*red*) and anti-EGFP immunolabeling (*green*) and nuclear staining (*blue*). **D** is a digitally zoomed in view of the boxed area in **C**. **E** and **G** show confocal images of anti-albumin labeled sections from wildtype (**E**) and *Cubn*^*+/del exon 1–6;EGFP*^ (**F**) mouse kidneys. **F** and **H** show images of the sections shown in **E** and **G** merged with anti-EGFP labeling. G, glomerulus.

Kidney sections from wildtype mice and mice heterozygous for *Cubn exon 1–6* deletion with an EGFP cassette insertion (*Cubn*^*+/del exon 1–6;EGFP*^) were immunolabeled with antibodies to cubilin and EGFP. In kidneys from wildtype mice, all proximal tubules displayed prominent and relatively uniform brush border immunolabeling with anti-cubilin IgG (Figure 1A). By contrast, the kidneys of *Cubn*^*+/del exon 1–6;EGFP*^ mice showed strong immunolabeling in the brush border regions of only a subset of proximal tubules (Figure 1B). Evaluation of EGFP immunolabeling in kidney sections from these mice revealed a similar discontinuous distribution, with some cells showing high levels of anti-EGFP immunofluorescence while adjacent cells had only weak fluorescence (Figure 1C). However, strikingly, the epithelial cells that had strong EGFP immunofluorescence displayed little or no cubilin immunolabeling (Figure 1D). Conversely, the proximal tubules that showed relatively low EGFP immunofluorescence displayed pronounced cubilin immunolabeling. Similar observations were made in the kidneys of female *Cubn*^*+/del exon 1–6;EGFP*^ mice (*data not shown*). Thus, in the proximal tubule cells of *Cubn*^*+/del exon 1–6;EGFP*^ mice, the level of EGFP immunofluorescence was inversely related to the level of cubilin immunofluorescence. These observations suggest that one of the two cubilin alleles in these heterozygous mice, either the targeted deletion/EGFP insertion allele or the wild-type cubilin allele, is suppressed while the remaining allele is active. Collectively, the findings suggest that the cubilin gene is subject to monoallelic inactivation in the kidney. The allelic inactivation appears to be stochastic in that adjacent proximal tubule cells could be found in which one cell was expressing high levels of EGFP and the other not. The fact that most proximal tubule cells in *Cubn*^*+/del exon 1–6;EGFP*^ kidneys were not completely devoid of EGFP immunofluorescence suggests that the inactivation process is not absolute.

### EGFP expressing, cubilin deficient proximal tubule cells display reduced albumin localization in Cubn^*+/del exon 1–6;EGFP*^ mice

Cubilin located on the brush border of renal proximal tubule cells mediates binding and endocytosis of albumin from the glomerular filtrate [12]. In kidneys of wildtype mice, albumin is localized on the apical/brush border regions of renal proximal tubules (Figure 1E and F). In *Cubn*^*+/del exon 1–6;EGFP*^ mice, pronounced albumin immunolabeling was apparent in the apical/brush border regions of a subset of renal proximal tubules that showed relatively low EGFP immunofluorescence (Figure 1G and H). By contrast, proximal tubules with strong anti-EGFP immunofluorescence showed little or no albumin immunolabeling in the brush border region. These findings suggest that the cubilin-deficient proximal tubules (i.e., those cells with strong anti-EGFP immunofluorescence) are unable to efficiently bind and endocytose albumin, which is consistent with other studies showing that cubilin deficiency leads to albuminuria [12,26].

### Expression of megalin and amnionless in the kidney of Cubn^*+/del exon 1–6;EGFP*^ mice

**Figure 2:**
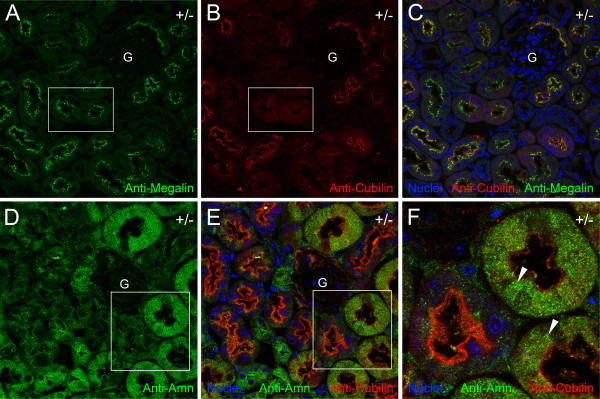
**Megalin expression is uniform whereas amnionless accumulates in cubilin-deficient renal proximal tubules of Cubn**^***+/del exon 1–6;EGFP***^**mice. A-C** show confocal images of a section of *Cubn*^*+/del exon 1–6;EGFP*^ mouse kidney labeled with anti-megalin IgG (**A**), anti-cubilin (**B**). **C** is a merged image of anti-megalin and anti-cubilin immunolabeling together with Draq5 nuclear staining (*blue*). Boxed areas in **A** and **B** highlight an example of a proximal tubule displaying strong anti-megalin immunolabeling and relatively weak anti-cubilin labeling. **D** shows a confocal image from a section of a *Cubn*^*+/del exon 1–6;EGFP*^ mouse kidney labeled with anti-amnionless (Amn). **E** is a merged image of the section of *Cubn*^*+/del exon 1–6;EGFP*^ mouse kidney shown in **D** labeled with anti-amnionless, anti-cubilin and the nuclear stain, Draq5 (*blue*). **F** is a digitally zoomed in view of the boxed areas in **D** and **E** showing a proximal tubule displaying strong anti-cubilin immunolabeling adjacent to two proximal tubules with little or no anti-cubilin labeling. *Arrowheads* indicate intracellular accumulations of Amn immunolabeling. G, glomerulus.

We next evaluated the expression of two cubilin-binding membrane proteins, megalin and amnionless, in proximal tubules of *Cubn*^*+/del exon 1–6;EGFP*^ mice. As shown in Figure 2A, anti-megalin immunolabeling was relatively uniform in the brush border regions of all proximal tubules. Furthermore, the relative levels of megalin immunolabeling were uniform among all proximal tubules, irrespective of the varied levels of anti-cubilin immunolabeling (Figure 2A and *B boxed areas*). Based on these findings, cubilin deficiency resulting from monoallelic inactivation in *Cubn*^*+/del exon 1–6;EGFP*^ mice apparently has no effect on the expression of megalin in the renal proximal tubule brush border. By contrast, immunofluorescence analysis of amnionless showed that in proximal tubules having little or no anti-cubilin labeling, amnionless accumulated within the proximal tubule cells as compared to proximal tubule cells having high levels of anti-cubilin labeling (Figure 2D-F). These findings are consistent with previous studies showing that cubilin prevents intracellular accumulation of amnionless [27].

### Expression of cubilin in the intestine

**Figure 3:**
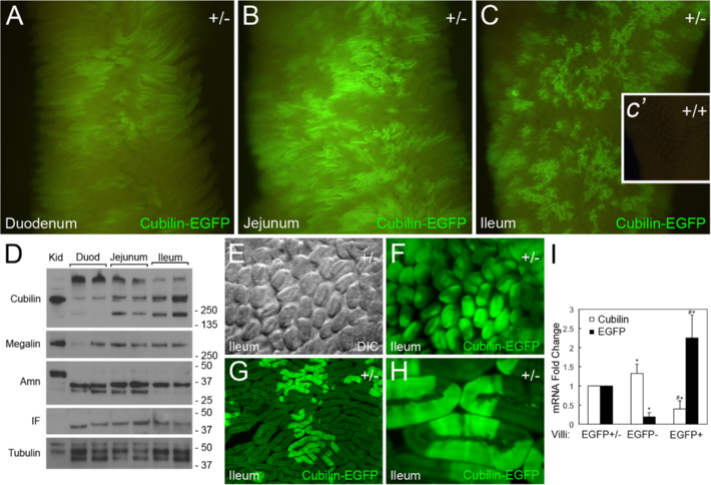
**Cubilin expression in the intestine of Cubn**^***+/del exon 1–6;EGFP***^**mice. A-C** show images of EGFP fluorescence in the luminal surface of whole mount segments of the duodenum (**A**), jejunum (**B**) and ileum (**C**) from a *Cubn*^*+/del exon 1–6;EGFP*^ mouse. Inset panel *c’* shows a segment of the ileum from a wildtype mouse subjected to epifluorscent imaging as in **A-C**. **D** shows an immunoblot analysis of detergent extracts of segments of the intestine and kidney using antibodies to cubilin, megalin, amnionless (Amn), intrinsic factor (IF) and tubulin. **E** is a DIC image of the luminal face of the small intestine (ileum) from a *Cubn*^*+/del exon 1–6;EGFP*^ mouse. **F** is an epifluorescence image showing EGFP fluorescence in the epithelial cells of the intestinal villi shown in **E**. Note the mosaic pattern of cubilin-EGFP fluorescence. **G** is a cross section of the small intestine from a *Cubn*^*+/del exon 1–6;EGFP*^ mouse showing cubilin-EGFP expression in patches of villi. **H** is a high magnification view of a cluster of villi showing segmental cubilin-EGFP expression in epithelial cells within individual villi. **I** is a qPCR analysis of cubilin and EGFP mRNA levels in microdissected intestinal villi segments (n=4 mice) that were either predominantly EGFP-positive, negative or both. *Asterisks* indicate that mRNA levels measured in EGFP-positive or EGFP-negative villi isolates were significantly different (p<0.05) from the levels in RNA prepared from villi containing both EGFP-positive and EGFP-negative cells. *Pound* signs indicate that mRNA levels measured in EGFP-positive villi were significantly different (p<0.05) from levels in EGFP-negative villi.

EGFP-fluorescence (hereafter referred to as cubilin-EGFP fluorescence) was analyzed in whole mounts of intestinal segments from *Cubn*^*+/del exon 1–6;EGFP*^ mice. Cubilin-EGFP fluorescence was detected in all segments of the small intestine, i.e., duodenum, jejunum and ileum (Figure 3).

Cubilin expression in the duodenum had not been previously reported. To substantiate this, anti-cubilin immunoblot analysis of intestinal extracts from wild-type mice was performed (Figure 3D). Similar to what has been observed in rat and canine ileal extracts [6,28], mouse ileum and jejunum extracts contained ~460 kDa monomer, multimer and ~200 kDa forms of cubilin. Cubilin was also detected in extracts of the duodenum, however the stoichiometry of the various immunoreactive forms in the duodenum was different from that of ileum and jejunum (i.e., levels of cubilin multimer > monomer > ~200 kDa forms). The cubilin membrane anchor, amnionless, was detected in all three segments of the small intestine (Figure 5D). The data indicates that multiple amnionless polypeptides are apparent in extracts of the kidney and intestine and that their stoichiometry differs between the two tissues. Amnionless polypeptides have been previously described as having Mr values of 35–50 kDa, which corresponds to the range of polypeptides we observe in the intestine and kidney. The basis for these different forms is not clear, but may represent different posttranslational or postendocytic modifications of the protein as has been speculated [29]. The cubilin ligand intrinsic factor was also observed in all three segments of the small intestine (Figure 5D). Megalin expression in the small intestine was also examined and highest levels were detected in the ileum and jejunum and lowest levels in the duodenum (Figure 3D).

In all three segments of the small intestine, cubilin-EGFP fluorescence was found in patches of intestinal villi, with regions of relatively strong fluorescence interspersed in areas of little or no fluorescence. Closer examination revealed that cubilin-EGFP fluorescence was in discrete segments of each villus (Figure 3E-H).

To test the hypothesis that cubilin is monoallelically expressed in the small intestine, we performed qPCR analysis on microdissected villi from the ileum of *Cubn*^*+/del exon 1–6;EGFP*^ mice that were predominantly either EGFP positive, EGFP negative or both. If cubilin expression were biallelic, then the mosaic pattern might be explained by there being two populations of cells, one with both alleles inactive (EGFP-negative) and the other with both alleles active (EGFP-positive). If this were the case then EGFP-positive cells would express cubilin and EGFP mRNAs at equivalent levels. Furthermore, the EGFP-negative cells would express neither transcript. However, as shown in Figure 3I, predominantly EGFP-negative cells were not only found to express cubilin mRNA but the levels were also significantly elevated as compared to the predominantly EGFP-positive population. Conversely, EGFP-negative enterocytes expressed significantly lower EGFP mRNA levels as compared to EGFP-positive cells (Figure 3I). Based on these findings the cubilin gene appears to be largely expressed from one allele in intestinal cells (i.e., monoallelic expression).

**Figure 4:**
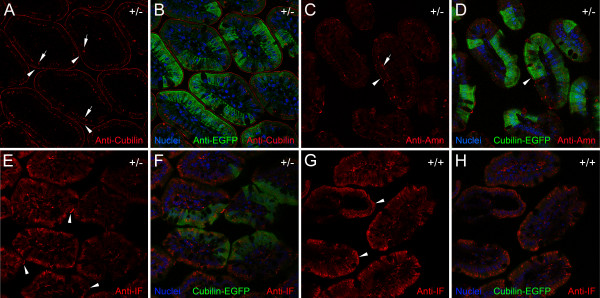
**Subcellular localization of cubilin, amnionless and intrinsic factor in ileal enterocytes. A** shows a confocal image from a section of a *Cubn*^*+/del exon 1–6;EGFP*^ mouse ileum labeled with anti-cubilin. **B** is a merged image of the section of *Cubn*^*+/del exon 1–6;EGFP*^ mouse intestine shown in **A** labeled with anti-cubilin and anti-EGFP together with Draq5 nuclear staining (*blue*). *Arrowheads* in **A** and **B** point to anti-cubilin label on the apical surfaces of enterocytes. *Arrows* in **A** and **B** point to anti-cubilin label in the cytoplasm of anti-EGFP-positive enterocytes. *Asterisks* in **B** indicate goblet cells devoid of anti-EGFP labeling and lacking apical anti-cubilin labeling. **C** shows a confocal image from a section of a *Cubn*^*+/del exon 1–6;EGFP*^ mouse ileum labeled with anti-amnionless. **D** is a merged image of the section of *Cubn*^*+/del exon 1–6;EGFP*^ mouse intestine in **C** showing cubilin-EGFP expression together with Draq5 nuclear labeling. *Arrowheads* in **C** and **D** point to anti-amnionless label on the apical surfaces of enterocytes. *Arrows* in **C** and **D** point to anti-amnionless label in the cytoplasm of EGFP-positive enterocytes. **E** shows a confocal image from a section of a *Cubn*^*+/del exon 1–6;EGFP*^ mouse ileum labeled with anti-intrinsic factor (IF). **F** is a merged image of the section of *Cubn*^*+/del exon 1–6;EGFP*^ mouse intestine in *E* together with cubilin-EGFP expression. *Arrowheads* in *E* point to anti-intrinsic factor IgG-reactive material present on the surfaces of both anti-EGFP-positive and anti-EGFP-negative enterocytes. **G** shows a confocal image from a section of a wildtype mouse ileum labeled with anti-intrinsic factor. **H** is a merged image of the section of the wildtype mouse intestine shown in **G** together with the image from the EGFP channel.

We next examined sections of small intestine from *Cubn*^*+/del exon 1–6;EGFP*^ mice after immunolabeling with antibodies to cubilin and EGFP. Anti-cubilin IgG-reactive material was apparent as a discrete layer on the apical surfaces of anti-EGFP-positive enterocytes as well as anti-EGFP-negative enterocytes (Figure 4A, arrowheads). In addition, anti-cubilin immunolabel was also detected within the cytoplasm of ileal enterocytes as punctate foci located on the apical side of the nuclei (Figure 4A, arrows). This cytoplasmic staining was present in both anti-EGFP-positive and anti-EGFP-negative enterocytes. Thus, despite EGFP-positive enterocytes having significantly lower expression of cubilin mRNA (Figure 3I), they appear to have similar cubilin protein levels compared to EGFP-negative enterocytes, which have higher expression of cubilin mRNA. These findings are consistent with the fact that cubilin is known to be secreted and taken up by enterocytes [30,31]. As such, enterocytes with the active wild-type cubilin allele might express and secrete cubilin, which could then bind to EGFP-positive enterocytes and result in similar levels of cubilin protein in all enterocytes.

### Amnionless in the intestine of Cubn^*+/del exon 1–6;EGFP*^ mice

We also performed anti-amnionless labeling of sections of small intestine from *Cubn*^*+/del exon 1–6;EGFP*^ mice. Amnionless immunolabeling was present on the cell surface and in the cytoplasm of EGFP-positive and EGFP-negative enterocytes (Figure 4C and D). There were no apparent differences in the relative levels or in the subcellular localization of amnionless between EGFP-positive and EGFP-negative enterocytes. This is in contrast to what was seen in the kidney, in which amnionless accumulated within EGFP-positive proximal tubule cells as compared to EGFP-negative proximal tubule cells (Figure 2F).

### Intrinsic factor in the intestine of Cubn^*+/del exon 1–6;EGFP*^ mice

Cubilin expressed by intestinal enterocytes mediates binding and endocytosis of intrinsic factor-cobalamin complex from the intestinal lumen [32]. Ileal segments from *Cubn*^*+/del exon 1–6;EGFP*^ mice were examined for immunolocalization of intrinsic factor in cubilin-EGFP-positive and cubilin-EGFP-negative enterocytes. As shown in Figure 4E and F, intrinsic factor immunolabeling was observed on the apical surfaces (Figure 4E, arrowheads) and within the cytoplasm of both EGFP-positive and EGFP-negative enterocytes. There was no apparent difference in the relative level of anti-intrinsic factor immunolabel in EGFP-positive and EGFP-negative enterocytes (Figure 4F). This was similar to the pattern of intrinsic factor immunolabeling observed in the wildtype intestine (Figure 4G and H). Based on these observations, the pattern of intrinsic factor uptake corresponds to anti-cubilin immunolabel found on all enterocytes (Figure 4A), regardless of the mosaic pattern of cubilin-EGFP or cubilin mRNA expression.

### Epigenetic regulation of cubilin

Several lines of evidence suggest that one of the two cubilin alleles may be inactivated epigenetically in the kidney and intestine: 1) EGFP expressed from the targeted cubilin allele displays a distinct mosaic pattern in the kidney and intestine, 2) proximal tubule cells expressing the targeted cubilin allele expressed little or no cubilin and displayed reduced binding of albumin, suggesting that the wild-type cubilin allele in these cells was inactive, and 3) EGFP-negative enterocytes express significantly higher levels of cubilin mRNA as compared to EGFP-positive cells in *Cubn*^*+/del exon 1–6;EGFP*^ mice. Inactivation of the cubilin allele might occur through epigenetic modifications that control the transition of chromatin from transcriptionally active to inactive state. Histone deacetylation and DNA methylation at CpG sequences are two types of epigenetic modifications that mediate transcriptional inactivation.

### Cubilin promoter lacks conserved CpG islands

To determine whether DNA methylation regulates cubilin transcription, we first analyzed the regions flanking the transcription start site of the mouse cubilin gene for CpG islands using the CpG island analysis feature of the UCSC Genome Browser and the EMBOSS CpGPlot algorithm [33,34]. Analysis of 5′ flanking sequences extending from approximately -20,000 to +3,500 relative to the transcription start site of the mouse cubilin gene did not detect any CpG islands (*data not shown*). A similar analysis of the human and rat cubilin genes using the CpG island analysis feature of the UCSC Genome Browser did not detect any CpG islands. These findings did not preclude the possibility that cubilin expression in these species might be regulated by non-CpG DNA methylation [35]. It is also possible that DNA methylation indirectly regulates cubilin transcriptional activation.

### Effects of 5Aza on cubilin in NRK and Caco-2 cells

**Figure 5:**
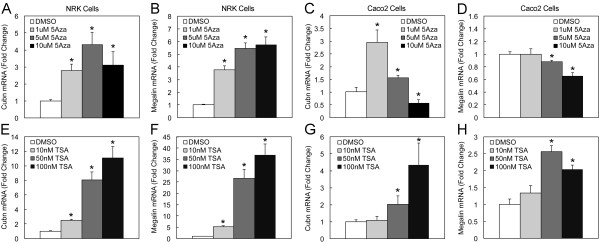
**5Aza and TSA elicit increases in cubilin expression in NRK and Caco-2 cells. A-D**, qPCR analysis of cubilin and megalin mRNA expression in RNA isolated from NRK and Caco-2 cells cultured for 60 h with three medium changes and 84 h with four medium changes, respectively, each containing the indicated doses of 5Aza. **E-H**, qPCR analysis of cubilin and megalin mRNA expression in RNA isolated from cells cultured for 24 h with the indicated doses of TSA. Cubilin and megalin mRNA expression data were normalized to GAPDH levels. *Asterisks* indicate that differences between mRNA levels in vehicle control and drug treatment groups were significant at p<0.05.

We therefore evaluated the effects of the DNA methylation inhibitor, 5-azacytidine (5Aza), on cubilin expression in renal NRK cells and intestinal Caco-2 cells. In NRK cells, 5Aza treatments augmented cubilin mRNA expression as measured by qPCR (Figure 5A). In Caco-2 cells, 1 and 5 μM 5Aza treatments augmented cubilin expression, but 10 μM 5Aza decreased cubilin expression (Figure 5C) as compared to the vehicle control. These findings suggested that DNA methylation was directly or indirectly regulating cubilin expression in cultured renal and intestinal epithelial cells. The effect of 5Aza treatment on the expression of megalin was also evaluated in these cells. 5Aza treatment stimulated megalin expression in NRK cells (Figure 5B), but inhibited megalin expression in Caco-2 cells (Figure 5D).

### Effects of TSA on cubilin in NRK and Caco-2 cells

We next evaluated the effects of the histone deacetylase (HDAC) inhibitor, trichostatin A (TSA), on cubilin mRNA expression in NRK and Caco-2 cells. TSA treatment elicited a concentration dependent increase in cubilin mRNA expression in both cell lines (Figure 5E and G). The magnitude of the effect of TSA on cubilin expression was generally greater than that achieved using 5Aza. TSA treatment also augmented megalin expression in both NRK and Caco-2 cells (Figure 5F and H).

### Effects of 5Aza and TSA on cubilin monoallelic expression in PRTCs

To determine whether allelic inactivation of the cubilin gene might be released by 5Aza or TSA treatment, we employed primary renal tubule cells (PRTCs) isolated from *Cubn*^*+/del exon 1–6;EGFP*^ kidneys. Following isolation of these cells, we consistently observed that <10% of the cultured PRTCs were EGFP-positive which is in contrast to the approximately 1:1 ratio of EFGP-positive to EGFP-negative cells in proximal tubules as assessed by immunofluorescence microscopy. The basis for this observation remains to be defined but it may be an indication that cubilin deficiency in PRTCs might reduce their migration out of tubules onto the culture plate, inhibit adhesion to the culture plate, or reduce cell proliferation *in vitro*.

**Figure 6:**
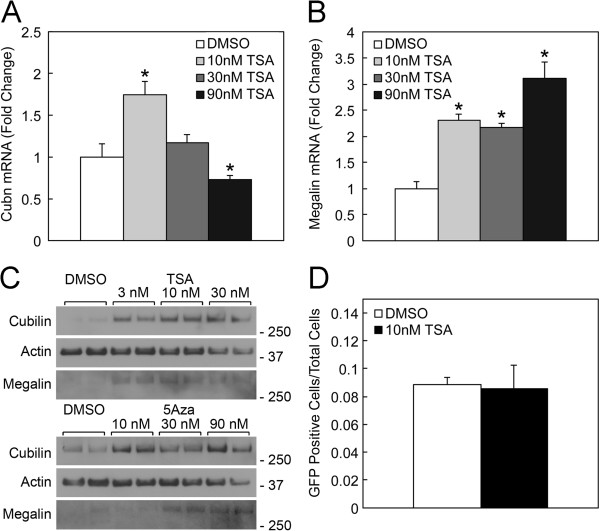
**TSA or 5Aza treatment does not have an effect on cubilin monoallelic expression in PRTCs. A** and **B**, qPCR analysis of cubilin and megalin mRNA expression in RNA isolated from PRTCs treated for 7 days with DMSO or with the indicated doses of TSA. **C**, shows immunoblot analysis of extracts of PRTCs treated for 7 days with vehicle or varying concentrations of TSA or 5Aza using antibodies to cubilin, megalin, and actin. **D** shows the number of anti-EGFP positive cells as a fraction of total cells counted in 21 imaged fields of DMSO-treated and 34 imaged fields of 10 nM TSA-treated PRTCs from three experiments. *Asterisks* indicate that differences between vehicle control and drug treatment groups were significant at p<0.05.

The mixed population of PRTCs (i.e., EFGP-positive and EGFP-negative PRTCs) isolated from *Cubn*^*+/del exon 1–6;EGFP*^ kidneys was treated with TSA. TSA treatment (10 nM) resulted in a ~2-fold increase in cubilin mRNA levels (Figure 6A), but the effect was reduced at higher concentration. TSA treatment also increased megalin mRNA levels (Figure 6B). Immunoblot analysis of extracts from TSA-treated PRTCs was also performed and the results showed that both cubilin and megalin protein expression was augmented by the treatment (Figure 6C). Furthermore, immunoblot analysis of extracts from 5Aza treated PRTCs was performed and the results showed that both cubilin and megalin protein expression was augmented by the treatment (Figure 6C).

We next reasoned that if 5Aza or TSA treatment were able to transform cubilin monoallelic expression to biallelic, then we should observe an increase in the number of anti-EGFP positive cells. We therefore determined the percent of EGF-positive cells in 21 imaged fields of DMSO treated PRTCs and in 34 imaged fields of TSA treated (10 nM) PRTCs. As shown in Figure 6D, TSA treatment of PRTCs did not elicit a significant change in the percentage of anti-EGFP positive PRTCs as compared to vehicle treated cells (also see Additional file 1). Similarly, PRTCs treated with 5Aza did not lead to an increase in the number of anti-EGFP positive cells as compared to vehicle treated cells (*data not shown*). In these 5Aza experiments, PRTC proliferation was inhibited by 5Aza treatment to a much greater extent than was observed for NRK cells. Since TSA or 5Aza treatment did not increase the number of EGFP expressing cells, we concluded that cubilin monoallelic expression was not effected by either treatment. Evaluating the effects of combined TSA and 5Aza was not possible due to the detrimental effects that the combined drugs had on PRTC growth. For this reason, we evaluated the effects of combined TSA and 5Aza treatment using NRK cells. The results showed that the stimulation of cubilin mRNA expression achieved by combined TSA and 5Aza treatment was not greater than that observed with TSA, which consistently elicited the greatest magnitude increase (*data not shown*).

### Epigenetic regulation of cubilin via PPARs

**Figure 7:**
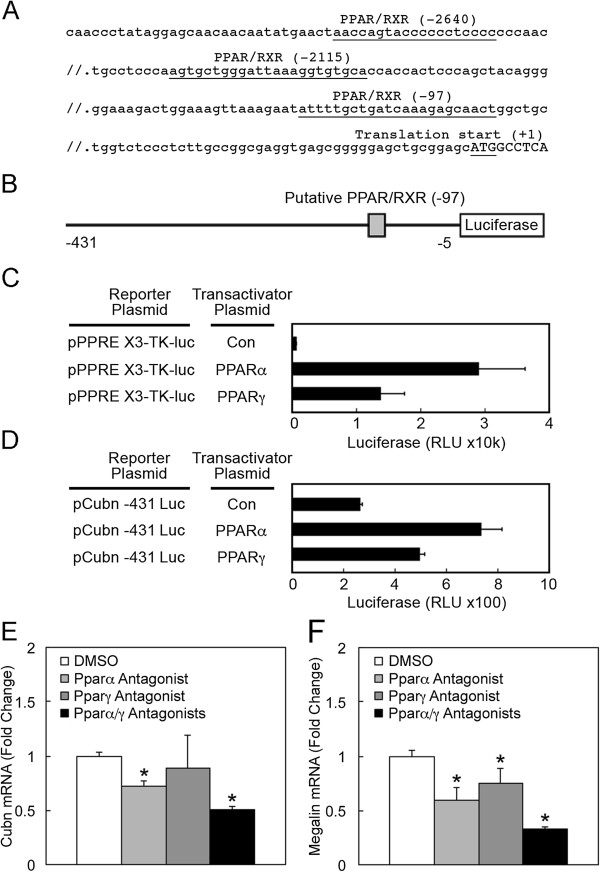
**PPAR regulation of cubilin expression. A**, upstream flanking sequence of the mouse cubilin gene (Cubn). Putative PPAR/RXR heterodimer binding sites, identified by Genomatix DiAlign, are indicated. Sequence elements are numbered relative to the translation start site (+1). **B**, representation of the pCub -431 Luc mouse Cubn promoter construct containing mouse Cubn proximal flanking sequence (-431 to -5), including the -97 putative PPAR/RXR binding element (shaded box), linked to a luciferase cassette. **C**, an established PPAR reporter construct is transactivated by PPARα and PPARγ in transfected BN cells. Reporter plasmid (pPPRE X3-TK-luc) was cotransfected with either a PPARα expression plasmid (pSG5 PPAR alpha), a PPARγ expression plasmid (pcDNA flag PPAR gamma) or a negative control plasmid (Con). Relative luciferase activity in lysates from transfected cells is shown. **D**, PPARα and PPARγ transactivate the mouse cubilin proximal promoter. Cotransfections were done with the pCub-431 Luc promoter plasmid and either PPARα, PPARγ, or a negative control plasmid (Con) as described for *C*. Relative luciferase activity in lysates from transfected cells is shown. **E** and **F**, qPCR analysis of cubilin (Cubn) and megalin mRNA in NRK cells cultured in the presence or absence of PPARα or PPARγ antagonists, GW6471 and GW9662 (each at 10 μM) for 24 h. *Asterisks* indicate p<0.05.

**Figure 8:**
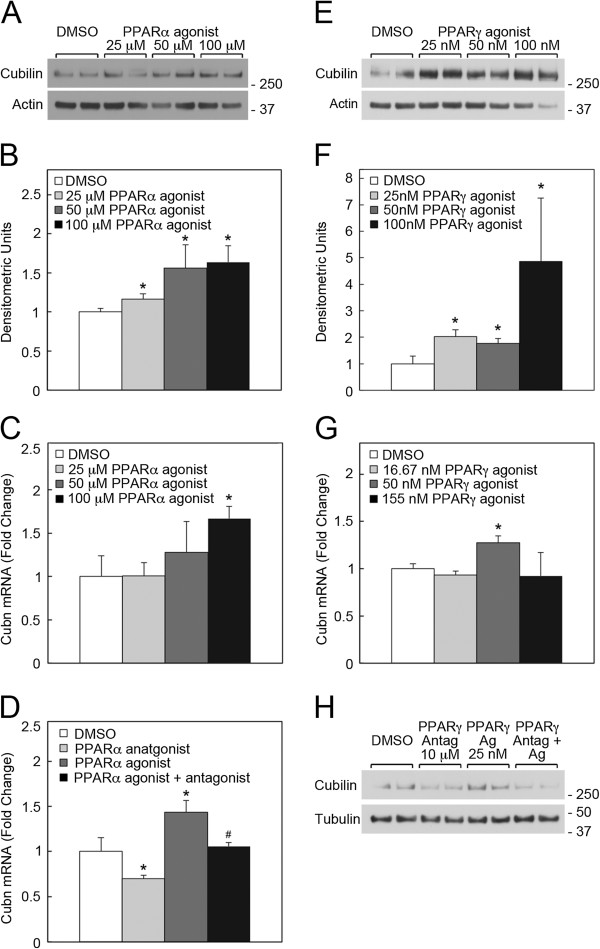
**PPARα and γ regulate cubilin expression in PRTCs. A** shows anti-cubilin and actin immunoblot analysis of detergent extracts of PRTCs treated with the PPARα agonist, Wy14643, at the indicated concentrations for 22 h. **B** shows densitometric analysis of three replicate anti-cubilin immunoblot experiments similar to **A**. **C** shows qPCR analysis of cubilin mRNA expression in PRTCs treated with the PPARα agonist, Wy14643, at the indicated concentrations for 22 h. **D** shows qPCR analysis of cubilin mRNA expression in PRTCs treated with either PPARα agonist (100 μM), PPARα antagonist, GW6471 (10 μM), or both for 22 h. **E** shows anti-cubilin and actin immunoblot analysis of detergent extracts of PRTCs treated daily with the PPARγ agonist, Rosiglitazone, at the indicated concentrations for 3 days. **F** shows densitometric analysis of three replicate anti-cubilin immunoblot experiments similar to *E*. **G** shows qPCR analysis of cubilin mRNA expression in PRTCs treated daily with the PPARγ agonist, Rosiglitazone, at the indicated concentrations for 3 days. **H** shows anti-cubilin and tubulin immunoblot analysis of detergent extracts of PRTCs treated twice with PPARγ antagonist, GW9662 (10 μM), PPARγ agonist, Rosiglitazone (25 nM), or both for 36 h. *Asterisks* indicate that differences between vehicle control and drug treatment groups were significant at p<0.05. *Pound* signs indicate that differences between agonist treatment alone and combined agonist with antagonist treatment groups were significant at p<0.05.

The above findings suggested that TSA and 5Aza treatment increased cubilin expression by increasing the transcription of the active cubilin allele. HDACs, which are inhibited by TSA, are known co-repressors of the transcription factors, Peroxisome Proliferator-Activated Receptor (PPAR) [36-39]. Conversely, PPAR co-activators increase histone acetylation, resulting in PPAR-mediated transcription [36,38-40]. Recent studies show that the expression of PPARγ is also regulated by DNA methylation [41] and histone deacetylation [39]. Furthermore, renal megalin expression is known to be regulated by PPARs [15]. We therefore tested whether epigenetic regulation of PPARs mediated the effects of 5Aza and TSA on cubilin and megalin expression. Computerized transcription factor binding site analysis identified three PPAR response elements conserved in human and mouse cubilin promoters (Figure 7). Analysis of cubilin promoter activity in cells transfected with a cubilin promoter-luciferase reporter found that cubilin transcription was increased by transfection of cells with PPARα and γ expression constructs (Figure 7B-D). Furthermore, endogenous cubilin (and megalin) mRNA expression in NRK cells was blocked by the PPARα and γ antagonists, GW6471 and GW9662 (Figure 7E and F). To further evaluate the role of PPARs in regulating cubilin expression, we utilized PRTCs. PPARα agonist, Wy14643, treatment of PRTCs resulted in a significant increase in cubilin protein levels over a range of agonist concentrations (Figure 8A). Cubilin mRNA levels were also significantly increased by PPARα agonist treatment at 100 μM concentration (Figure 8C). By contrast, PPARα antagonist, GW6471, significantly decreased endogenous cubilin mRNA expression and also inhibited the increased cubilin expression achieved by agonist treatment (Figure 8D). PPARα agonist and antagonist treatments resulted in similar reciprocal changes in the mRNA levels of *Acadl*, a known PPARα-responsive gene [42,43] (Additional file 2). Treatment of PRTCs with the PPARγ agonist (Rosiglitazone) also resulted in a significant increase in cubilin protein levels (Figure 8E). Rosiglitazone at 50 nM concentration also elicited a modest, but significant increase in cubilin mRNA expression (Figure 8G). Furthermore, the PPARγ antagonist, GW9662, inhibited the increased cubilin expression achieved by Rosiglitazone treatment (Figure 8H). Together, these findings indicate that, similar to megalin [15], cubilin is a gene regulated by both PPARα and γ.

**Figure 9:**
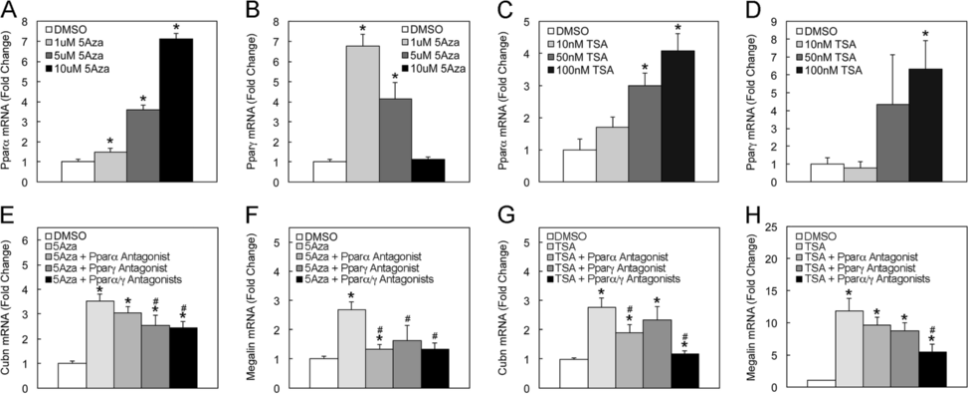
**5Aza and TSA elicit increases in PPARα/γ expression, and PPAR antagonists block 5Aza and TSA-mediated increase in cubilin expression. A**-**B**, qPCR analysis of PPARα and γ mRNA expression in RNA isolated from NRK cells cultured for 60 h with 3 medium changes each containing the indicated doses of 5Aza. **C** and **D**, qPCR analysis of PPARα and γ mRNA expression in RNA isolated from NRK cells cultured for 24 h with the indicated doses of TSA. **E** and **F**, qPCR analysis of cubilin and megalin was performed on RNA from NRK cells treated for 36 h with 2 medium changes each containing 5 μM of 5Aza and then a 24 h treatment with 5Aza-free medium containing PPAR antagonists (each at 10 μM). **G** and **H**, qPCR analysis of cubilin and megalin mRNA in RNA isolated from NRK cells cultured for 24 h with TSA alone (100 nM) or TSA (100 nM) plus PPAR antagonists (each at 10 μM). Cubilin, megalin and PPARα and γ mRNA levels were normalized to GAPDH. *Asterisks* indicate that differences between mRNA levels in vehicle control and drug treatment groups were significant at p<0.05. *Pound* signs indicate that differences between mRNA levels in drug treatment groups alone and drug treatment with PPAR antagonists were significant at p<0.05.

We next evaluated the effects of 5Aza and TSA on the expression of PPARα and γ in NRK cells. As shown in Figure 9A, 5Aza treatment elicited a dose dependent increase in PPARα mRNA expression. 5Aza also augmented PPARγ expression (Figure 9B) at low concentrations but not at the highest concentration tested (10 μM). Similarly, TSA treatment of NRK cells also produced a concentration dependent increase in PPARα mRNA levels (Figure 9C). TSA also increased PPARγ expression at the highest concentration tested (100 nM) (Figure 9D).

The observations that TSA and 5Aza increased PPARα and γ mRNA levels suggested the possibility that cubilin upregulation by TSA and 5Aza resulted from increased expression of PPAR. We therefore evaluated the effects of PPARα and γ antagonists on TSA and 5Aza induction of cubilin expression. As shown in Figure 9E, the upregulation of cubilin by 5Aza was inhibited by PPARγ antagonist as well as combined PPARα and γ antagonist treatments. *Acadl*, a known PPARα responsive gene [42,43], was also increased by 5Aza treatment and the increase was inhibited by PPARα antagonist treatment (Additional file 2B). These findings suggested that the 5Aza-induced expression of cubilin is dependent on 5Aza-induced expression of PPARγ. Parallel studies showed that 5Aza-mediated induction of megalin expression was also dependent on 5Aza-induced expression of both PPARα and γ (Figure 9F).

The effects of PPARα and γ antagonists on TSA-induced cubilin expression were also evaluated. Results showed that cubilin upregulation by TSA was inhibited by PPARα antagonist as well as combined PPARα and γ antagonist treatments (Figure 9G). The fact that combined PPARα and γ antagonist treatment produced a greater magnitude decrease than either antagonist alone suggested that TSA-induced expression of cubilin is dependent on TSA-induced expression of both PPARα and γ. Parallel studies showed that TSA-induced expression of megalin was also dependent on TSA-induced expression of both PPARα and γ (Figure 9H). *Acadl* levels were also increased in response to TSA treatment and the increase inhibited by PPAR antagonist treatment (Additional file 2C).

**Figure 10:**
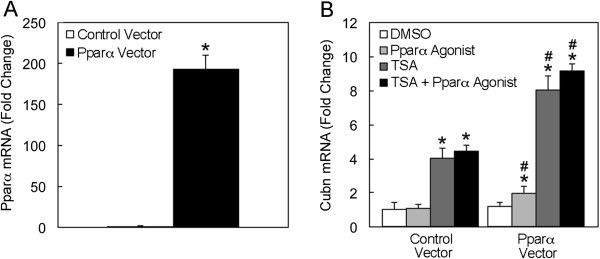
**TSA-mediated increase in cubilin expression involves increases in both PPAR transcription and activation. A**, qPCR analysis of PPARα mRNA levels in NRK cells transfected with PPARα expression construct or a control vector. *Asterisk* in A indicates that differences between mRNA levels in control and PPARα were significant at p<0.05. **B**, qPCR analysis of cubilin mRNA levels in NRK cells transfected with PPARα expression construct or a control vector and treated for 24 h with either PPARα agonist, Wy14643 (100 μM) , TSA (100 nM) or TSA (100 nM) plus PPARα agonist (100 μM). *Asterisks* indicate that differences between mRNA levels in vehicle control and drug treatment groups were significant at p<0.05. *Pound* signs indicate that differences between mRNA levels in control vector and PPARα vector transfected cells with the same treatments were significant at p<0.05.

We next tested whether the increased transcription of PPARs was sufficient to induce cubilin expression or whether the epigenetic modifiers also influenced the activation state of PPAR. NRK cells were transfected with a PPARα expression construct, which greatly increased levels of PPARα mRNA (Figure 10A). However, the increased PPARα levels alone did not increase cubilin mRNA levels (Figure 10B). Similarly, the addition of PPARα agonist alone did not alter cubilin expression (Figure 10B). Yet the combination of PPARα overexpression and PPARα agonist treatment caused a significant increase in cubilin mRNA expression. These findings indicate that under normal conditions PPARs are fully active in NRK cells and the addition of an agonist alone does not increase cubilin expression. Thus, both PPARα upregulation and a PPARα agonist are required to augment cubilin expression. When control transfected cells were treated with TSA there was a 4-fold increase in cubilin expression (Figure 10B). The addition of agonist to these cells did not elicit any further increase (Figure 10B). By contrast, PPARα overexpression combined with TSA treatment caused an 8-fold increase in cubilin expression. As with controls, the addition of agonist to these cells did not significantly increase the level of cubilin expression (Figure 10B). These findings indicate that the TSA mediated increase in cubilin expression not only involves an increase in PPAR transcription but also PPAR activation.

## Discussion

Here we present evidence that the cubilin gene undergoes allelic inactivation. The evidence includes findings showing that cubilin expression in the kidneys of mice heterozygous for targeted cubilin deletion/EGFP insertion is mosaic such that some proximal tubule cells display active expression of EGFP (i.e., the targeted allele) as well as suppressed expression of the wild-type cubilin allele, while other proximal tubule cells display the inverse pattern. Mosaic expression of cubilin was also observed in all three segments of the small intestine, with cubilin-EGFP fluorescence found in discrete segments of each villus. Additional support for enterocytes displaying allelic inactivation of the cubilin gene came from qPCR studies of *Cubn*^*+/del exon 1–6;EGFP*^ mice showing that cubilin mRNA levels were significantly reduced in isolated regions of the small intestine expressing EGFP as compared to non-EGFP expressing regions. At the protein level, unlike what was seen in the kidney, enterocytes displayed both expression of the cubilin allele bearing the targeted deletion/EGFP insertion and contained immunologically detectable cubilin. Since enterocytes have been shown to release cubilin in association with extracellular surfactant-like particles [31], which bind to the apical portion of the cell [30], it is reasonable to expect that cubilin expressed in some enterocytes from the wild-type cubilin allele is being released and taken up via cubilin binding receptors (e.g., megalin and/or amnionless) expressed by enterocytes in which the wild-type cubilin allele is suppressed. Furthermore, this form of cubilin appears to be capable of endocytosis of its ligand, intrinsic-factor cobalamin complex, since intrinsic factor uptake was similar in enterocytes with suppressed wild-type cubilin allele and those with an active wild-type cubilin allele.

Unlike what was observed in the kidney where amnionless accumulates intracellularly in cubilin-deficient cells, the consequences of cubilin deficiency on amnionless trafficking in intestinal cells was not clear. Based on our intestinal data, we speculated that a secreted form of intestinal cubilin might act in a non-cell autonomous manner to prevent the accumulation of amnionless in enterocytes having lower endogenous cubilin expression. It is also important to point out that the intestine appears to express multiple cubilin and amnionless isoforms, which have different stoichiometries in the kidney (Figure 3D). Therefore, it is possible that in the intestine, cubilin and amnionless trafficking to the apical cell membrane are not interdependent.

The finding that 5Aza and TSA treatments were unable to release the suppression of the silenced cubilin allele suggests that DNA methylation and histone deacetylation may not be the only mechanisms of regulation of cubilin monoallelic expression. Indeed, diverse mechanisms exist to mediate allelic inactivation including interplay of DNA modifications by DNA methylation and modifications of the histone proteins by acetylation, methylation, SUMOylation or phosphorylation [44,45]. Additionally, imprinted monoallelic expression of certain gene clusters is in part mediated by noncoding RNAs under the control of methylation of imprint control elements [44]. Intriguingly, the cubilin gene is located in the mouse chromosome 2 proximal region, which is one of several chromosomal regions known to contain genes that undergo parental imprinting during development [46,47]. Our observation that groups of adjacent proximal tubule cells shared the same inactive cubilin allele suggests that the allelic inactivation is inherited clonally from a progenitor that underwent imprinting during development. Further research will be required to define the precise mechanisms regulating cubilin monoallelic expression.

Since cubilin monoallelic expression was unaltered by 5Aza and TSA treatments, we concluded that the increased cubilin mRNA levels were due to effects of these agents on the transcriptionally active cubilin allele. Both histone deacetylation and DNA methylation are associated with PPAR repression [39,41]. In fact, HDAC1 and 3 are known PPARγ co-repressors [48-50]. Conversely, PPAR co-activators, such as CBP/p300 and SRC-1, possess histone acetylase activity required for chromatin remodeling to allow PPAR-mediated transcription [39,40,51,52]. Furthermore, recent findings show that the cubilin coreceptor, megalin, is a PPAR-responsive gene and its expression is augmented through inhibition of histone deacetylation [15,16]. In light of these studies, we recognized the possibility that the observed effects of TSA and 5Aza on cubilin might also involve PPAR induction. This led us to discover that the observed effects of 5Aza and TSA on cubilin expression were dependent on PPARα and γ induction. Specifically, we showed that cubilin was a PPARα and γ responsive gene, that the expression of PPARα and γ was inducible by 5Aza and TSA alone, and that the effects of TSA and 5Aza on cubilin expression were dependent on increased expression of PPARα and γ. Similarly, we showed that TSA and 5Aza-induced megalin expression was dependent on increased expression of PPARα and γ.

While TSA and 5Aza treatments augmented PPAR and cubilin mRNA levels, no significant increase in cubilin levels was achieved by overexpressing PPARα in the absence of PPARα agonist. Therefore, the effects of TSA and 5Aza on cubilin mRNA levels cannot be attributed to increased PPAR mRNA levels alone. We inferred that, in addition to increasing PPAR expression, 5Aza and TSA treatments must also lead to PPAR activation. The underlying mechanism for the apparent agonist-independent effects of TSA on induction of PPAR-dependent transcription of cubilin remains to be established. TSA and/or 5Aza might augment levels of endogenous PPAR agonists, cause demethylation of sites on the cubilin gene, or reduce histone occupancy of the cubilin promoter. Any of these effects might be sufficient for promoting PPAR action in an agonist-independent manner. Since HDACs are known co-repressors and HATs are co-activators of PPARs [39], it is reasonable to expect that inhibiting HDAC activity will shift PPARs towards a more active state. In fact, HDAC inhibitors have been reported to act as atypical PPAR agonists [38] and studies have shown that inhibition of HDACs stimulates transcription of PPAR responsive genes [48,53]. Therefore, it is likely that increased acetylation of histones associated with the cubilin promoter in response to HDAC inhibitor treatment is sufficient to promote PPAR-driven cubilin expression.

Our findings that cubilin and megalin expression is regulated by DNA methylation and histone deacetylation raise questions as to whether these genes might be predisposed to inactivation by disease-associated increases in DNA methylation and histone deacetylation. DNA methylation is a major contributing factor to various disease-related processes, such as tumorigenesis, atherogenesis and diabetic nephropathy [54-56] (including glomerular and interstitial fibrosis [57]). Global DNA hypermethylation is associated with inflammation and increased mortality in chronic kidney disease [58] and chronic inflammation has even been implicated as a driving factor associated with increased DNA methylation in diseases such as chronic gastritis and gastric cancer [59,60]. Furthermore, the inflammatory cytokine, IL-6, exerts an impact on epigenetic changes in cells via regulation of DNA methyltransferase [61]. Histone deacetylation catalyzed by HDACs also contributes to the pathogenesis of various diseases including gastric and colorectal cancer [62,63], renal disease such as polycystic kidney disease [64] and macrophage infiltration and fibrotic changes associated with tubulointerstitial injury [65].

HDAC inhibitors appear to have significant therapeutic potential in kidney disease [66]. For example, a number of studies have demonstrated efficacy of TSA in ameliorating renal injury in mice following unilateral ureteral obstruction [65], nephrotoxic serum nephritis [67] and in lupus pathogenesis [68]. In light of findings presented herein, it is possible that drugs that inhibit HDACs might ameliorate renal disease by releasing epigenetic suppression of PPARs, cubilin and megalin. Intriguing new findings from rodent studies highlight the potential reno-protective benefits of increased megalin expression on early phase renal injury in responses to protein overload [15]. Specifically, the studies showed that megalin expression in rats is decreased by BSA overload and that augmenting megalin expression in rats by PPARγ agonist treatment correlated with a reduction in BSA-induced proteinuria. Effects of PPAR agonist treatments on renal expression of the albumin receptor, cubilin, were not evaluated in those studies. Thus, it was not clear whether the mechanistic basis for the observed effects involved PPAR agonist induced changes in cubilin expression. Our studies demonstrate that cubilin, like megalin, is under PPAR transcriptional regulation and suggest that the amelioration of protein overload-induced albuminuria by PPAR agonists observed in other studies is mediated by augmented levels of cubilin.

## Conclusions

Cubilin expression is epigenetically regulated by at least two processes. The first process involves allelic inactivation that is not reversible by inhibiting DNA methylation and histone deacetylation. The second process involves transcriptional regulation of cubilin by PPAR transcription factors that are themselves regulated by DNA methylation and histone deacetylation.

## Methods

### Animals

All studies involved the use of 1–6 month old male mice heterozygous for cubilin exon 1–6 deletion with an EGFP cassette insertion (*Cubn*^*+/del exon 1–6;EGFP*^) [17] or age/sex matched wildtype littermates. Mouse experimentation was conducted with approval from the IACUC.

### Antibodies

Goat anti-cubilin IgG (A20) was purchased from Santa Cruz Biotechnology, Inc. (Santa Cruz, CA), rabbit anti-EGFP IgG was from Abcam (Cambridge, MA), goat anti-albumin was obtained from Bethyl Laboratories, Inc. (Montgomery, TX), mouse anti-human α-tubulin was purchased from Sigma-Aldrich (St. Louis, MO) and rabbit anti-actin was from Abcam. Rabbit anti-human intrinsic factor serum was generously provided by Dr. David H. Alpers (Washington University School of Medicine, St. Louis, MO). Rabbit anti-porcine megalin (rb6286) was described previously [69]. Antiserum to ammnionless (rb4156/57) was prepared by immunizing rabbits with synthetic multiple antigenic peptide containing amino acid residues 165–178 (accession number NM_001108061.1). The resulting antiserum reacted with a single ~45 kDa polypeptide in mouse kidney extracts and ~35 kDa and 30 kDa bands in intestinal extracts and an ~30 kDa band in extracts of E8.5 mouse embryo extracts and rat BN cells (Figure 3D and *data not shown*). Donkey anti-goat and donkey anti-rabbit-Alexa Fluor (488 or 568) conjugates were purchased from Invitrogen (Carlsbad, CA).

### Tissue procurement and immunofluorescence

Following euthanization, animals were perfused first with phosphate-buffered saline (PBS) and then with 4% paraformaldehyde, PBS. Tissues were dissected and further fixed by immersion in 4% paraformaldehyde PBS for 12 h. For kidney immunohistochemical analysis, ~0.25 cm thick strips of cortex tissue were isolated. For small intestine immunohistochemical analysis, ~10 cm long segments of duodenum (extending distally from the fundus of the stomach), jejunum (beginning ~5 cm from the end of duodenum) and ileum (ending ~2 cm superior of the cecum) were isolated. Isolated tissues were embedded in paraffin and sectioned at 6 μm thickness. Tissue sections were incubated with primary antibodies diluted in PBS containing 3% BSA, washed with PBS and incubated with Alexa Fluor conjugated secondary antibodies. Nuclei were stained using Draq5 (Cell Signaling Technology, Danvers, MA). Labeled sections were analyzed using a Leica SP5 confocal microscope (Leica Microsystems Inc., Exton, PA)using the 63× objective or a Zeiss Axio M2 microscope using the 40× objective. Whole mount images of unlabeled intestine EGFP-fluorescence were taken by a Leica MZ FLIII microscope at ~5× magnification.

Intestinal villi from *Cubn*^*+/del exon 1–6;EGFP*^ mouse ileum were micro-dissected under GFP light at 10× magnification using Leica MZ FLIII microscope. Villi that appeared predominantly EGFP-positive or EGFP-negative as well as a random sampling of both were separately isolated using dissection scissors in Hank’s buffered salt solution (HBSS) (Thermo Scientific) containing 4% FBS. Villi were then briefly span down, the supernatant removed and RNA extracted from the samples using the RNeasy Plus Mini Kit (QIAGEN, Valencia, CA). qPCR was performed as described below.

### Immunoblot analysis

Unfixed segments of small intestine were homogenized in 1% Triton X-100, 0.5% Tween20, 0.5 M NaCl, 50 mM Hepes, pH 7.5 containing a protease inhibitor cocktail (Complete-mini, EDTA-free, Roche, Germany) using a Polytron-aggregate (Brinkmann Instruments, Switzerland). Extracts were subjected to centrifugation at 100 K × *g* for 30 min at 4°C. Protein concentration in extracts was quantified using Pierce BCA Protein Assay Kit (Rockford, IL). Equal amounts of protein from the extracts were loaded onto NuPAGE 4-12% polyacrylamide gradient, Bis-Tris gels in the presence of SDS (no reducing agent). Following electrophoresis, the proteins were transferred onto PVDF membranes, which were blocked with 5% non-fat milk in TBS and probed with primary and secondary antibodies diluted in TBS containing 0.5% Tween 20 and 5% non-fat milk. Chemiluminescent detection of bound antibodies was achieved using the Pierce ECL Western Blotting Substrate.

### Cell culture

NRK-52E (NRK) (ATCC no. CRL-1571) and Caco-2 (ATCC no. HTB-37) cells were cultured in DMEM (high glucose) with 10% fetal bovine serum, Glutamax (Life Technologies, Grand Island, NY), non-essential amino acids, 100 units/ml penicillin, and 100 units/ml streptomycin (Thermo Scientific, Rockford, IL), and maintained at 37°C and 5% CO_2_. For trichostatin A (TSA) treatments, cells were plated at 1.0 × 10^4^ cells/cm^2^ for 6 h, and subsequently treated for 24 h with 10, 50 or 100 nM TSA (Sigma, St. Louis, MO) (281.6 pM final DMSO concentration in medium). For 5-Azacytidine (5Aza) treatments, NRK cells were plated at 5 × 10^3^ cells/cm^2^ for 6 h and subsequently treated with 1, 5 or 10 μM 5Aza (Sigma) (281.6 pM final DMSO concentration in medium) for 60 h with three media changes at 0, 12 and 36 h. For Caco-2 cells, 5Aza treatments were for 84 h with four media changes at 0, 12, 36 and 60 h. To evaluate the effects of PPAR transcription factor antagonists on TSA treated cells, TSA treated NRK cells were incubated with PPARα antagonist (GW6471), PPARγ antagonist, (GW9662) (Sigma), or both, each at 10 μM for 24 h. To evaluate the effects of PPAR transcription factor antagonists on 5Aza treated cells, NRK cells were treated for 36 h with 5Aza and incubated with 5Aza-free medium containing PPAR antagonists, each at 10 μM for 24 h. In studies evaluating the effects of transgenic PPARα, NRK cells were transiently transfected with pSG5 PPAR alpha plasmid [70] (plasmid 22751; Addgene, Cambridge, MA) using Lipofectamine LTX & PLUS Reagent (Invitrogen) according to manufacturer’s instructions. Nearly confluent NRK cells, grown in six-well plates, were transfected with 1 μg pSG5 PPAR alpha plasmid or a control plasmid using Lipofectamine LTX at a 1:3 mass:volume ratio for ~18 h. The cells were then allowed to grow for 24 h in complete medium and treated with TSA or vehicle as described above.

Primary renal tubule cells (PRTCs) were isolated and cultured as described previously [71,72]. Briefly, mouse renal cortices were dissected and minced in ice-cold dissection solution of Hank’s buffered salt solution (HBSS) (Thermo Scientific) supplemented with 10 mM glucose, 5 mM glycine, 1 mM alanine and 15 mM HEPES pH 7.4. The minced fragments were transferred to a dissection solution containing 96 μg/ml soybean trypsin inhibitor, 1 mg/ml type 1 collagenase and 0.05% type 2 collagenase, and digested for 30 min at 37°C. After digestion, the mixture was passed through a 250 μm pore size nylon sieve and the flow-through material passed through an 80 μm pore size nylon sieve. Proximal tubules (PT) retained on the 80 μm sieve were resuspended with 37°C HBSS solution containing 1% BSA and then subjected to centrifugation for 5 min at 170 × *g*. The PT pellet was resuspended in 1:1 mixture of DMEM:F12 culture medium containing 15 mM HEPES and 2 mM L-glutamine (Thermo Scientific) supplemented with 9% FBS, 50 nM hydrocortisone, ITS (BD Biosciences, San Jose, CA), sodium pyruvate, non-essential amino acids, 100 units/ml penicillin, and 100 units/ml streptomycin (Thermo Scientific), and then plated and maintained at 37°C and 5% CO_2_. PRTCs were cultured for 4 days before treatment with a medium change every other day. PRTCs were then treated with 5Aza or TSA or DMSO (vehicle)-containing medium for additional 7 days. On day 7, PRTCs were used for protein or RNA extraction using the RNeasy Plus Mini Kit (QIAGEN, Valencia, CA) or fixed in 4% paraformaldehyde PBS with 1% Triton-X100 for 20 min for immunofluorescent microscopy.

For PPAR agonist and antagonist studies, semi-confluent PRTC cultures were treated with PPAR alpha agonist, Wy14643 (Cayman Chemical Company, Ann Arbor, MI), PPAR alpha antagonist, GW6471 (Sigma) or vehicle DMSO only at the indicated concentrations for 22 hrs in complete media containing 3%FBS. Protein or RNA was extracted from independent experiments as already described.

### qPCR

RNA from cells or tissue was isolated using the RNeasy Plus Mini Kit (QIAGEN, Valencia, CA) and quality assessed on an Agilent Bioanalyzer. cDNA was prepared from 0.25-1 μg total RNA using the iScript cDNA Synthesis Kit (Bio-Rad, Hercules, CA) according to manufacturer instructions. qPCR was performed using iQ SYBR Green Supermix (Quanta BioSciences, Inc., Gaithersburg, MD) reagents and a C1000 Thermal Cycler (Bio-Rad). The relative values for each gene were determined using the cycle thresholds and normalized to reference genes. The following qPCR primers were used: rat *Cubilin* forward, 5′-TGGGAGCTGCGTCTATGATT-3′ and rat *Cubilin* reverse, 5′- AAGGAAGTTGCCGGAAGAGA-3′ (NM_053332.2); rat *Megalin/Lrp2* forward, 5′- ATCTGTGCTCCAGGTCCAAA-3′ and rat *Megalin/Lrp2* reverse, 5′-ATTGAGGCAGGTGAACTGGA-3′ (NM_030827.1); rat *Ppar*α forward, 5′- GGCCAAGAGAATCCACGAAG-3′ and rat *PPAR*α reverse, 5′- ACAAAAGGCGGATTGTTGCT-3′ (NM_013196.1); rat *Ppar*γ forward, 5′- GGTGAAACTCTGGGAGATCCT-3′ and rat *PPAR*γ reverse, 5′- CATGGTAATTTCTTGTGAAGTGCT-3′ (NM_013124.3); rat *Gapdh* forward, 5′- GTGCCAGCCTCGTCTCATA-3′ and rat *Gapdh* reverse, 5′- AGGTCAATGAAGGGGTCGTT-3′ (NM_017008.3); rat/mouse *Rn18s* forward, 5′- CGCCGCTAGAGGTGAAATTCT-3′ and rat/mouse *Rn18s* reverse, 5′- CGAACCTCCGACTTTCGTTCT-3′ (NR_046237.1); human *Cubilin* forward, 5′- CCTGAACTGCGAATGGACTC-3′ and human *Cubilin* reverse, 5′- CATCACCCACTCGAAACTCG-3′ (NM_001081.3); human *Lrp2* forward, 5′- GATTGGGCTGCTTCACGATT-3′ and human *Lrp2* reverse, 5′- ATGGCAAGTCCAAACGGATG-3′ (NM_004525.2); human *Gapdh* forward, 5′- ATGTTCGTCATGGGTGTGAA-3′ and human *Gapdh* reverse, 5′- GGTGCTAAGCAGTTGGTGGT-3′ (NM_002046.4); mouse *Cubilin* forward, 5′-ATTTTCTCTGGGGTTTTGTTAC-3′ and mouse *Cubilin* reverse, 5′-TAAGTTTCCCTCCTCCGTAG-3′ (NM_001081084.2); mouse *Megalin/Lrp2* forward, 5′-TGACTGCGGAGACATGAGTG-3′ and mouse *Megalin/Lrp2* reverse, 5′-CACAGACCCAGTGTTGTGGA-3′ (NM_001081088.1); mouse *Gapdh* forward, 5′-CAGCCTCGTCCCGTAGACA-3′ and mouse *Gapdh* reverse, 5′-CAACAATCTCCACTTTGCCACT-3′ (NM_008084.2); mouse *βactin* forward, 5′-AACCGCTCGTTGCCAATA-3′ and mouse *βactin* reverse, 5′-CGGGACCTGACAGACTACCTC-3′ (NM_008084.2); and *GFP* forward, 5′-CAACAGCCACAACGTCTATATCATG-3′ and GFP reverse, 5′-ATGTTGTGGCGGATCTTGAAG-3′ (U50963.1) [73].

### Cubilin promoter luciferase transfection assays

BN cells for luciferase reporter assays were passaged in complete medium (MEM with 10% fetal bovine serum, Glutamax (Life Technologies, Grand Island, NY), non-essential amino acids, 100 units/ml penicillin, and 100 units/ml streptomycin (Thermo Scientific) as described previously [8]. Prior to transfection, BN cells were plated at 0.5 ×10^5^ cells/cm^2^ in 24-well culture dishes in complete medium and grown overnight. Control transfections were performed with PPARα (pSG5 PPAR alpha), PPARγ (pcDNA flag PPAR gamma; Plasmid 8895, Addgene) [74] or pcDNA3.1(+) (Life Technologies, Grand Island, NY) expression plasmids in combination with a PPAR responsive luciferase reporter plasmid (PPRE X3-TK-luc; Plasmid 1015, Addgene) [75]. Experimental transfections were conducted with PPARα, PPARγ or pcDNA3.1(+) expression plasmids in combination with a cubilin promoter luciferase plasmid (pCub -431 Luc) created by insertion of a mouse cubilin promoter fragment (-431 to -5, relative to the translation start site) into the pGL3-Basic luciferase reporter plasmid (Promega, Madison, WI). Transfections were performed in triplicate with GenePORTER 2 (Genlantis, San Diego, CA) using 1.5 μg of each plasmid (3 μg total) and 15 μl GenePORTER 2 per transfection. Transfections proceeded for 24 h after which cells were lysed and extracts prepared for luciferase assay with the BD Monolight Enhanced Luciferase Assay Kit (BD Biosciences, San Jose, CA) according to manufacturer recommendations. Luciferase activity was measured using a Monolight 2012 Luminometer (BD Biosciences).

### Statistical analysis

Data are presented as mean ± SD of 3 replicates, representative of at least 3 independent experiments. Two-tailed Student’s t-tests were used to compare control and treatment groups.

## Competing interests

The authors declare that they have no competing financial interests.

## Authors’ contributions

OA designed the experimentation, performed tissue procurement, immunofluorescence, immunoblot analysis, cell transfections and qPCR. JLB performed cubilin promoter analysis and cubilin promoter luciferase transfection assays. SCK assisted with cell culture. BTS was responsible for generation of the cubilin knockout mouse strain and early phenotypic analysis. WSA contributed to the experimental design, the presentation and interpretation of results, and coordinated the project and writing of the manuscript. All authors read and approved the final manuscript.

## Supplementary Material

Additional file 1 **A-C, show confocal images of anti-EGFP and anti-cubilin labeled renal proximal tubules cells isolated from the cortex of *Cubn+/del exon 1–6;EGFP *mouse kidneys and treated for 24 h with DMSO vehicle. D**-**F**, show confocal images of anti- EGFP and anti-cubilin labeled cells isolated from the renal cortex of *Cubn+/del exon 1–6;EGFP* mouse kidneys and treated for 24 h with TSA (10 nM). Nuclei (*blue*) were stained using Draq5. Click here for file

Additional file 2 **A, qPCR analysis of *Acadl *mRNA expression in PRTCs treated with PPARα antagonist, GW6471 (10 μM), PPARα agonist (100 μM), or both for 22 h. B**, qPCR analysis of *Acadl* was performed on RNA from NRK cells treated for 36 h with 2 medium changes each containing 5 μM of 5Aza and then a 24 h treatment with 5Aza-free medium containing PPAR antagonists (each at 10 μM). **C**, qPCR analysis of *Acadl* mRNA in RNA isolated from NRK cells cultured for 24 h with TSA alone (100 nM) or TSA (100 nM) plus PPAR antagonists (each at 10 μM). Click here for file
