# Supplementary figures and images for: Cubilin expression is monoallelic and epigenetically augmented via PPARs
Source: BMC Genomics. 2013 Jun 18;14:405. doi: 10.1186/1471-2164-14-405 (PMC3706236; doi:10.1186/1471-2164-14-405)

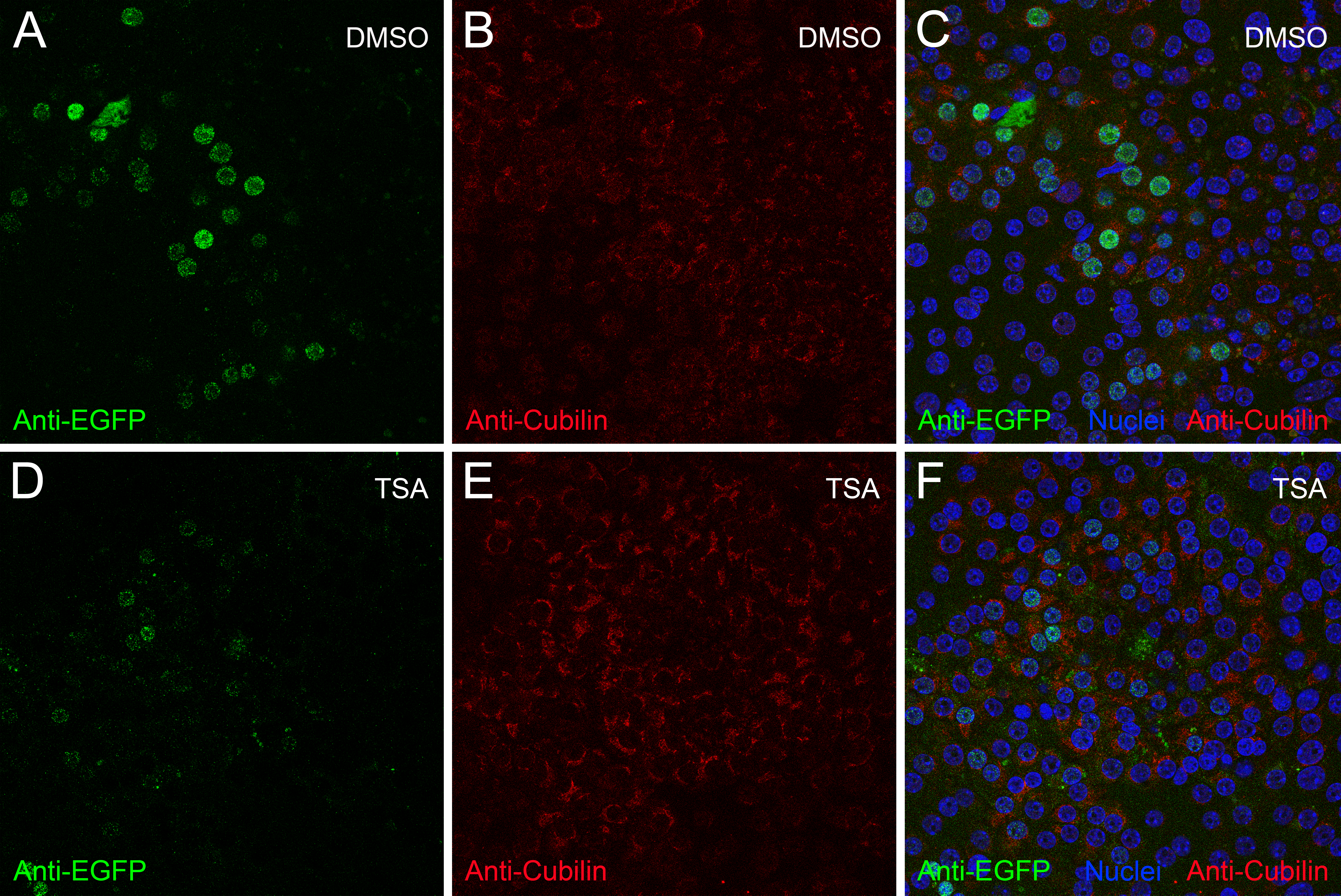

Supplement: Additional file 1 — A-C, show confocal images of anti-EGFP and anti-cubilin labeled renal proximal tubules cells isolated from the cortex of Cubn+/del exon 1–6;EGFP mouse kidneys and treated for 24 h with DMSO vehicle. D-F, show confocal images of anti- EGFP and anti-cubilin labeled cells isolated from the renal cortex of Cubn+/del exon 1–6;EGFP mouse kidneys and treated for 24 h with TSA (10 nM). Nuclei (blue) were stained using Draq5. [file 1471-2164-14-405-S1.tif]

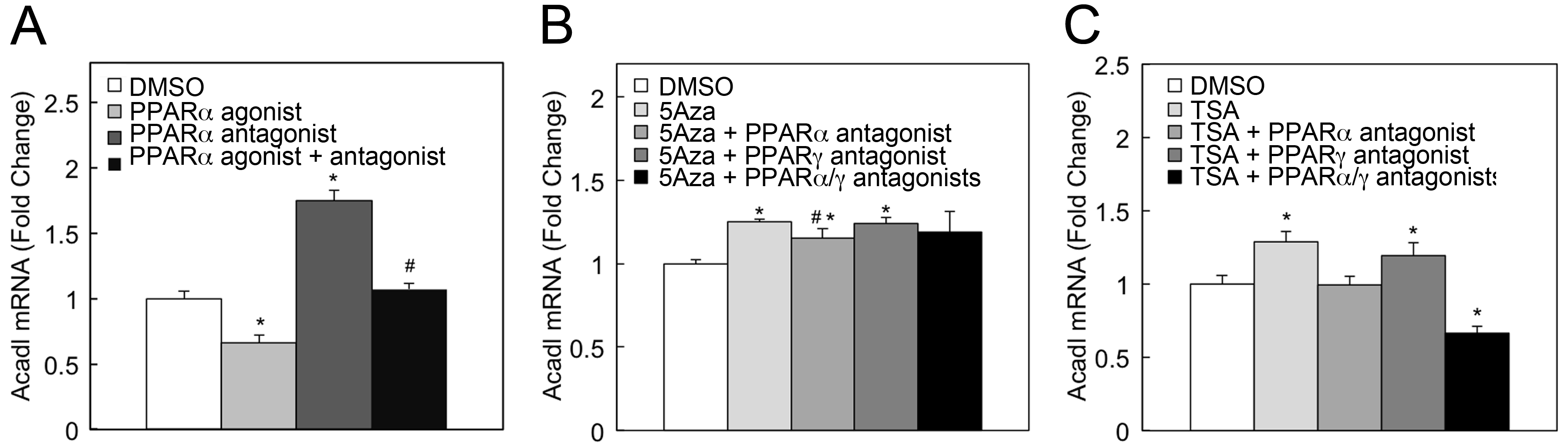

Supplement: Additional file 2 — A, qPCR analysis of Acadl mRNA expression in PRTCs treated with PPARα antagonist, GW6471 (10 μM), PPARα agonist (100 μM), or both for 22 h. B, qPCR analysis of Acadl was performed on RNA from NRK cells treated for 36 h with 2 medium changes each containing 5 μM of 5Aza and then a 24 h treatment with 5Aza-free medium containing PPAR antagonists (each at 10 μM). C, qPCR analysis of Acadl mRNA in RNA isolated from NRK cells cultured for 24 h with TSA alone (100 nM) or TSA (100 nM) plus PPAR antagonists (each at 10 μM). [file 1471-2164-14-405-S2.tif]
